# Effects of COVID-19 on cognition and mood after hospitalization and at 2-month follow-up

**DOI:** 10.3389/fpsyg.2023.1141809

**Published:** 2023-05-26

**Authors:** Manca Peskar, Boštjan Šimunič, Luka Šlosar, Saša Pišot, Kaja Teraž, Mladen Gasparini, Rado Pišot, Uros Marusic

**Affiliations:** ^1^Institute for Kinesiology Research, Science and Research Centre Koper, Koper, Slovenia; ^2^Biological Psychology and Neuroergonomics, Department of Psychology and Ergonomics, Faculty V: Mechanical Engineering and Transport Systems, Technische Universität Berlin, Berlin, Germany; ^3^Department of Health Sciences, Alma Mater Europaea – ECM, Maribor, Slovenia; ^4^Faculty of Sports, University of Ljubljana, Ljubljana, Slovenia; ^5^Department of General Surgery, General Hospital Izola, Izola, Slovenia

**Keywords:** COVID-19 recovery, acute respiratory syndrome Coronavirus-2, cognitive function, cognitive impairment, MOCA, trail-making test

## Abstract

A plethora of evidence links SARS-CoV-2 infection with concomitant cognitive dysfunction, which often persists weeks to months after the acute stages of illness and affects executive function, attention, memory, orientation, and movement control. It remains largely unclear which conditions or factors exacerbate the recovery. In a cohort of *N=*37 Slovenian patients (5 females, aged *M* = 58, SD = 10.7 years) that were hospitalized because of COVID-19, the cognitive function and mood states were assessed immediately after discharge and 2-months later to investigate the early post-COVID recovery changes. We assessed the global Montreal Cognitive Assessment (MoCA), Simple and Choice Reaction Times, executive functions (Trail-Making Test – TMT-A and TMT-B), short-term memory (Auditory Verbal Learning Test – AVLT), and visuospatial memory. We monitored depressive and anxiety symptoms and applied general self-efficacy and cognitive complaints questionnaires. Our results showed a global cognitive impairment (MoCA, *Z* = 332.5; *p* = 0.012), poorer performance on executive functions (TMT-A, *Z* = 188; *p* = 0.014; and TMT-B, *Z* = 185; *p* = 0.012), verbal memory (AVLT, *F* = 33.4; *p* < 0.001), and delayed recall (AVLT7, *F* = 17.1; *p* < 0.001), and higher depressive (*Z* = 145; *p* = 0.015) and anxiety (*Z* = 141; *p* = 0.003) symptoms after hospital discharge compared to 2-month follow-up, indicating that SARS-CoV-2 may transiently impair cognitive function and adversely affect the mood. No improvement in MoCA was observed in 40.5% of the patients at follow-up, indicating possible long-term effects of COVID-19 on global cognitive performance. Medical comorbidities (*p* = 0.035) significantly predicted the change in MoCA score over time, while fat mass (FM, *p* = 0.518), Mediterranean diet index (*p* = .0.944), and Florida Cognitive Activities Score (*p* = 0.927) did not. These results suggest that the patients’ medical comorbidities at the time of SARS-CoV-2 infection could importantly contribute to the acute impairment of cognitive function and stress the importance of systemic implementation of countermeasures to limit the negative consequences on public health.

## 1. Introduction

As of March 28, 2023, there had been over 683 million recorded cases of COVID-19, with over 656 million survivors (Elflein, 2023). Despite a relatively high survival rate, the unfavorable consequences on patients’ health and well-being had been observed long after the acute disease stage during which the patients typically report poor concentration, sleep difficulty, excessive fatigue, as well as episodes of delusion and hallucinations (Taquet et al., 2021). However, the main cause of hospitalization and mortality after the infection with the Severe Acute Respiratory Syndrome Coronavirus-2 (SARS-Cov-2) which clinically manifests as COVID-19 disease, are respiratory difficulties (Lleó and Alcolea, 2020).

Several mechanisms had been implicated in the symptom manifestation. Possible mechanisms of the central nervous system damage have been identified in the brains of hamsters and humans deceased due to COVID-19, namely a disruption of the brain–blood barrier, activation of the microglia, and loss of hippocampal neurogenesis. The absence of such brain changes in patients deceased from other conditions than COVID-19 suggests that these changes may be specific to SARS-CoV-2 infection (Klein et al., 2021). The brain regions found to be especially affected by the infection were the ones associated with olfactory function, motor coordination, memory, and learning. Another post-mortem study of patients deceased from COVID-19 reports the presence of ischemic lesions and signs of neuroinflammation in the brainstem (Matschke et al., 2020). The virus could trigger a severe cytokine-mediated inflammatory response leading to brain damage and cognitive impairment. High levels of cytokines can cause the so-called “sickness behavior” syndrome, characterized by impaired concentration, reduced motivation, motor slowing, and depression (Dantzer et al., 2008). The inflammatory response that accompanies the development of COVID-19 is manifested by the increased levels of several inflammatory markers (Peiris et al., 2003; Huang et al., 2020) shown to promote cognitive decline, such as interleukin-1β and tumor necrosis factor-α (Dantzer et al., 2008) and these inflammatory mediators may persist long after the viral clearance (Peiris et al., 2003). Endothelial dysfunction in COVID-19 patients undergoing rehabilitation has also been associated with the pathogenesis of cognitive impairment (Moretta et al., 2022).

Studies investigating persisting or long-term symptomatic effects report that the majority of COVID-19 patients have a good recovery from the respiratory tract symptoms, however, changes in their mental health status reflected as cognitive impairment, neurological disturbances, and psychiatric disorders may persist long after other respiratory symptoms have subsided (Mazza et al., 2020, 2021; Benedetti et al., 2021). At discharge from the hospital, deficits of global cognitive function, such as executive function, attention, memory, orientation, movement control, and affective disorders, such as depressive and anxiety symptoms, have been observed in varying portions between 15 and 80% of the sampled COVID-19 patients (Almeria et al., 2020; Helms et al., 2020; Alemanno et al., 2021; Bonizzato et al., 2021; Daroische et al., 2021). Furthermore, the accumulated data suggest that cognitive symptoms which outlive the acute phase of the disease may occur irrespective of the experienced severity of the symptoms during the acute COVID-19 infection. Specifically, cognitive dysfunction and/or mood disturbance have been observed in patients at 2 to 12 months after the acute infection who survived severe or hospitalization-demanding disease progression (Méndez et al., 2021; Pistarini et al., 2021), in patients experiencing mild-to-moderate symptom severity during the acute COVID-19 infection (de Matos et al., 2021; Del Brutto et al., 2021), and surprisingly also in patients, whose disease progression was completely asymptomatic (Amalakanti et al., 2021). Impairments of cognition as assessed by MoCA were also detected in younger, that is middle-aged COVID-19 patients, who were free of any neurological disease and received no mechanical ventilation or oxygen supplementation while hospitalized (Solaro et al., 2021) The research into this post-COVID syndrome or long-COVID has repeatedly recognized cognitive dysfunction as one of the most frequently occurring symptoms identified in about 70% of patients (Cirulli et al., 2020; Bliddal et al., 2021; Davis et al., 2021; Ziauddeen et al., 2021). It should however be noted that the initial illness severity might impact the severity of persisting issues (Whitaker et al., 2021).

Several risk factors for a more severe disease progression and consequently longer recovery have thus far been recognized. Across the globe, increasing age, sex (male), the presence of comorbidities, such as cardiovascular disease or diabetes, and obesity were more likely to result in more severe COVID-19 conditions (Abrahim et al., 2020; Fu et al., 2020; Gao et al., 2020; Kompaniyets et al., 2021). In the US, a retrospective study (Sallis et al., 2021) also discovered that patients whose lifestyle was consistently inactive had a greater risk of hospitalization, admission to the intensive care unit, and death due to COVID-19 than patients who were consistently meeting physical activity guidelines. This notion suggested that especially in the elderly, cognitive-motor leisure-time or daily activities, such as walking a dog (Brown and Rhodes, 2006) or gardening (Nicklett et al., 2016), could importantly contribute to meeting physical activity guidelines and in turn, promote favorable post-acute recovery (Morley, 2020). In addition, the leisure time activities, such as playing chess, could benefit the COVID-19 recovery process by providing cognitive stimulation (Lillo-Crespo et al., 2019). To our knowledge, however, the relationship between engaging in daily cognitive activities and cognitively recovering from COVID-19 has not been investigated. Another factor associated with an increased risk for severe COVID-19 infection and mortality is poor metabolic health (Morys and Dagher, 2021) which can be affected by the dietary lifestyle decisions of an individual. Mediterranean diet by being high in antioxidants and anti-inflammatory properties (Estruch, 2010; Nani et al., 2021) provides metabolic benefits (Papadaki et al., 2020) and has been proposed as a promising method for attenuating the severity of COVID-19 infection and improving disease-related outcomes in healthy and diabetic population (Maiorino et al., 2020; Angelidi et al., 2021). The link between the Mediterranean diet and COVID-19, however, remains poorly investigated. We were interested to know whether the Mediterranean diet index could predict cognitive recovery after COVID-19 infection.

To better understand the COVID-19-related cognitive symptoms and mood disturbances, as well as their aftermath of recovery, we assessed for the first time the global and specific cognitive functions as well as the mood in a Slovenian cohort sample of COVID-19 patients who were admitted to the hospital due to a severe SARS-CoV-2 infection. Our first aim was to assess the patients at the time of discharge from the hospital (post-COVID) and after a 2-month post-discharge recovery period (post-Recovery) to monitor early recovery changes from the time after acute infection. We hypothesized that improvements in cognitive function and mood will be observed at post-Recovery compared to post-COVID in most participants, however, in a smaller portion of up to 30% of participants, we expected to observe persisting symptomatology. To understand which factors might contribute to a non-favorable outcome or be considered risk factors for disease progression, our second aim was to identify the variables that could predict short-term recovery. Here, our goals were to (i) validate the evidence obtained in other cultural backgrounds to the Slovenian population where we hypothesized that comorbidities and obesity are likely to negatively affect disease recovery; and to (ii) investigate the predictive power of two additional variables, specifically the scores on the Florida Cognitive Activities Scale (Schinka et al., 2005) and Mediterranean diet index (Sotos-Prieto et al., 2015), which have not received a great deal of scientific attention yet in terms of their association with COVID-19 recovery. For the latter, we hypothesized they might help predict the post-COVID-19 recovery.

## 2. Materials and methods

### 2.1. Participants

Participants enrolled in the study were recruited upon admission to the General Hospital Izola, Slovenia. All procedures were performed in accordance with the Declaration of Helsinki and were reviewed and approved by the Hospital’s ethics committee (application number: 1/21). Participants were first approached by a physician who explained the study protocol. Inclusion criteria were ≥ 18 years of age, signed informed consent, and completed hospital treatment after a positive polymerase chain reaction (PCR) nose swab test on the SARS-CoV-2 virus. The exclusion criteria were a positive PCR test on the SARS-CoV-2 virus upon discharge from the hospital, major injuries/damage to the musculoskeletal system (disability), and the inability to follow instructions while performing the tests. Out of 43 initially enrolled patients, 39 (5 females; aged *M* = 58.5; *SD* = 10.6 years) attended the measurements at both time points, however, additional two participants were excluded as they failed to complete all the tests, leading to the final sample of *N =* 37 patients. Demographic, health-related data, and other patient characteristics are presented in Table 1. The raw data collected are to be made available upon request (see *Data Availability Statement*).

**Table 1:** Demographic and health-related patient data.

	*N* (%)	*M*	*SD*	Range
Patients	37 (100)			
Fraction female	5 (13.5)			
Dyspnea at admission	25 (67.6)			
Oxygen saturation level < 90% at admission	33 (89.2)			
Received mechanical ventilation/intubation	0 (0)			
Age (years)		58.2	10.7	[34, 79]
Education (years)		12.8	2.2	[6, 19]
Height (cm)		175.4	8.7	[156, 194]
Weight (kg)		99.2	16.9	[74, 146]
Hospitalization (days)		7.0	4.9	[1, 30]
Including the intensive care unit	2 (5.4)			
Fat Mass (%)		30.2	10.8	[13.7, 58.2]
Obesity^1^	22 (59.5)			
Fraction Female	2 (9.1)			
Fraction with comorbidity/ies	18 (48.6)			
Comorbidity count				
Hypertension	16 (43.2)			
Diabetes Type II	7 (18.9)			
Psychiatric disorder	3 (8.1)			
Coronary disease	2 (5.4)			
Asthma	2 (5.4)			
Heart failure	1 (2.7)			
Atrial fibrillation	1 (2.7)			
Chronic kidney disease	1 (2.7)			
Chronic obstructive pulmonary disease	1 (2.7)			

1 Classification as obese was based on the percentage of fat mass (FM) obtained by bioelectrical impedance analysis; a cut off-score of 25.8% for men and 37.1% for women were considered (Macek et al., 2020).

### 2.2. Procedure

Participants were tested on 2 separate occasions; the first measurement point was on the 10th day after their last positive polymerase chain reaction COVID-19 test result (post-COVID), and the follow-up measurement was done 60.8 ± 2.52 (range: 55–65) days later (post-Recovery). All measurements were performed in the same room in the General Hospital Izola during morning hours. This study was part of a larger project in which other measures, such as functional capabilities tests, body composition, as well as blood and hair samples, were obtained. The protocol has been registered on ClinicalTrials.gov and details can be obtained under the identifier NCT04860206. Performing the tests reported here took 1 h, however, the whole testing session took 2 h. The tests and questionnaires were performed on both measurement days in the order presented below; for exceptions see “Subjective assessment and questionnaire”.

#### 2.2.1. Neurocognitive assessment

The *Montreal Cognitive Assessment* (MoCA; Nasreddine et al., 2005) screening tool for cognitive impairment was used to obtain a measure of global cognitive functioning. The MoCA assesses several cognitive domains, namely short-term memory, visuospatial abilities, executive functions, attention, concentration, working memory, language, and orientation to time and place. A score of <26 points is indicative of mild cognitive impairment. A total score ranges between 0 and 30 points.

The *Auditory verbal learning test* (AVLT; van der Elst et al., 2005) was used to assess verbal memory and a 30-min delayed recall. A list of 15 words (list A) is read, followed by an immediate (A1–5) and delayed (A7) recall by a participant. The same list has to be repeated following a distracting word list (A6). A total score reported for A1-5 is an average of the first five attempts, whereas for the A6 and A7 is the sum of the recalled items in each attempt. The scores range from 0 to 15. Norms are taken from Ivnik et al. (1990).

The *Trail-making test* (TMT; Reitan, 1958; Tombaugh, 2004) was used to assess the speed of visual search and executive function. In TMT-A and TMT-B, 25 randomly distributed encircled numbers or numbers and letters on a sheet of paper must be sequentially connected with a single line in an increasing and alternating fashion, if applicable. The scores for each part represent the time (in seconds) until completion. Normative data by Tombaugh (2004) are considered.

The computerized *Simple* (sRT) and *Choice reaction times* (cRT) known as the Deary-Liewald task (Deary et al., 2011) were recorded using the PsyToolkit platform (Stoet, 2010, 2017). In the sRT task, a single white square is presented in the center of the screen against the blue background. Whenever a black “X” appears within the square, a subject must respond as quickly as possible by pressing a spacebar key on a standard keyboard with the index finger of their dominant hand. Eight practice trials preceded the 20 test stimuli, which were then averaged for the final sRT score. Similarly, in the cRT task, four white squares ordered in a row are presented in the center of the screen. A black “X” appears in one of the squares per trial, and participants must indicate the correct answer by pressing one of the four keys, each corresponding to one of the spatial positions of the squares on the screen. From the leftmost to the rightmost square position, the following keys had to be pressed, respectively: the “z” key with the left middle finger, the “x” key with the left index finger, the “,” key with the right index finger, and the “.” key with the right middle finger. After eight practice trials, 40 test stimuli were sequentially presented and the response times to the correct positions averaged for the final score. Both speed and accuracy of the responses were encouraged. The scores are expressed in milliseconds [ms].

The *Corsi Block-Tapping task* (Corsi, 1972) obtained through the PsyToolkit (Stoet, 2010, 2017) was used to assess the visuospatial working memory. A participant is instructed to repeat the previously observed sequence of flashing squares by clicking on them in the same order as presented before. With each iteration, the sequence is becoming longer, starting with 2 and increasing by one each time. The score reflects the longest correctly reproduced sequence and ranges between 2 to 9.

#### 2.2.2. Subjective assessment and questionnaires

The *Beck’s Depression Inventory* (BDI; Beck et al., 1961) and *Beck’s Anxiety Inventory* (BAI; Beck, 1988) were used to obtain measures of depressive and anxiety symptoms, respectively. The scores for both BDI and BAI range between 0 and 63 points. Cognitive symptoms that usually accompany depression were assessed using the 6-item *British Colombia Cognitive Complaints Scale* (BC-CCI; Iverson and Lam, 2013). The scores range between 0 and 18 points, and the higher scores indicate the more severely expressed symptoms. Lastly, the 10-item *General Self-Efficacy Scale* (GSE; Jerusalem and Schwarzer, 1992) was used to obtain the measure of optimistic self-believes to cope with a variety of demands in life. The scores range between 10 and 40 points, and the higher scores indicate higher levels of optimistic self-believes.

From the 28-item Mediterranean lifestyle (MEDLIFE) questionnaire (Sotos-Prieto et al., 2015), the first block of items (15 in total) was used to derive data on the overall adherence to the Mediterranean diet. Zero or one point is given for each item and the higher number represents a more Mediterranean-like diet. The total score is obtained by summing up the points of all items and ranges between 0 and 15 points. Mediterranean diet index (MEDLIFE DI) was obtained at post-COVID and pertained to the 1 year before the COVID-19 infection.

To assess daily performed activities, the 25-item Florida Cognitive Activities Scale (Schinka et al., 2005) was used. It assesses the level of engagement in cognitive activities expressed in a unit over time (e.g., per day/week/month). Higher scores indicate greater activity levels. The questionnaire was applied at post-Recovery and pertained to the period of 2 months between hospital discharge and the follow-up assessment. The scores range between 1 and 100 points.

Subjective perception of the mental/cognitive and affective recovery at post-Recovery was assessed using the following question: “If you assign your pre-COVID-19 infection [insert: mental/cognitive or mood] state a 100%, at how many % do you feel you currently stand?”

### 2.3. Data analysis

This study was a within-subject design with factor *time* treated as the repeated measure (post-COVID, post-Recovery). Data were analyzed using IBM SPSS Statistics Version 26. All post-COVID – post-Recovery variable pairs were first checked for normality using Skewness, Kurtosis, and Shapiro–Wilk statistics. At post-COVID, deviations from normal distribution were detected in cRT, TMT-A, TMT-B, BDI, BAI, GSE, and BC-CCI, while at post-Recovery deviations from normality were observed in sRT, CORSI, MoCA, TMT-B, BDI, BAI, GSE, and BC-CCI. The variables pairs in which data of at least one measurement were not normally distributed, were analyzed using the non-parametric related samples Wilcoxon signed-ranks Test (reporting *Mdn, Z-statistics, value of p*). Alternatively, the recovery effects on AVLT1-5, AVLT6, and AVLT7 showed no violation of normality and were analyzed using the parametric repeated measures analysis of variance (ANOVA; time as a within-subject factor; reporting *M* (*SD*), and effect size using Cohen’s D). Alpha level was kept at 0.05.

Delta MoCA (ΔMoCA) was computed as a relative difference between post-Recovery and post-COVID MoCA scores and treated as a dependent variable in multiple linear regression. Variables used to predict ΔMoCA in a stepwise forward fashion at 0.05 alpha level were: (1) comorbidity; value 1 was assigned to participants with any diagnosis described previously (see *2.1. Participants)*, and 0 was assigned to the rest; (2) FM; (3) MEDLIFE DI; and (4) Florida Cognitive Activities Scale. No predictor showed deviation from normality as assessed by Shapiro–Wilk, Skewness, and Kurtosis statistics. Age was not chosen as a predictor due to significant correlations with comorbidity (*p* = 0.049, *r* = 0.33), FM (*p* = 0.015, *r* = −0.40), and MEDLIFE DI (*p* = 0.006, *r* = 0.45). No other correlations among the predictors were significant.

## 3. Results

### 3.1. Neurocognitive tests and questionnaires

Wilcoxon Signed-Ranks Tests revealed that the median scores for MoCA, TMT-A, TMT-B, BDI, and BAI at post-Recovery differed significantly from the median scores at post-COVID. while no differences between the two time points were observed for the sRT, cRT, CORSI, BC-CCI, and GSE variables. Table 2 provides an overview of the results. The Supplementary Table S1 summarizes the fraction of patients at post-COVID and post-Recovery whose scores were sub-normative or indicated cognitive impairments or mild-to-severe mood symptom severity.

**Table 2:** Results of the post-COVID to post-Recovery non-parametric related-samples analyses.

	Post-COVID				Post-recovery				Test Statistic (*Z*)	Sig. (*p*)
Variable	*M*	*SD*	*Mdn*	Range	*M*	*SD*	*Mdn*	Range		
MoCA	24.6	2.6	25	[19, 29]	25.7	2.7	26	[16, 30]	332.5	0.012*
sRT	315	44	307	[257, 415]	305	36.5	297	[254, 399]	241	0.148
cRT	593	120	607	[430, 1,075]	590	89.6	603	[413, 758]	364	0.850
CORSI	4.4	1.1	4	[3, 7]	4.6	1.2	5	[3, 6]	210	0.363
TMT-A	39.4	16.6	34.6	[18, 83]	33.1	9.7	29.8	[18, 59]	188	0.014*
TMT-B	101	54.3	86.5	[32, 257]	90.7	61.8	71.1	[35, 300]	185	0.012*
BDI	8.4	6.5	7	[1, 29]	6.5	6.5	3	[0, 24]	145	0.015*
BAI	11.5	9.6	7	[0, 37]	7.8	9.9	3	[0, 36]	141	0.003*^#^
BC-CCI	3.1	3.4	2	[0, 13]	3.4	4	2	[0, 14]	196.5	0.881
GSE	35.6	3.8	36	[26, 40]	34.4	5.1	36	[16, 40]	222	0.429

Mdn, median; MoCA, Montreal Cognitive Assessment; sRT, Simple Reaction Time; cRT, Choice Reaction Time, CORSI, Corsi Block Tapping Task; TMT-A, Trail-Making Test A; TMT-B, Trail-Making Test B; BDI, Beck Depression Inventory; BAI, Beck Anxiety Inventory; BC-CCI, British Columbia Cognitive Complaints Inventory; GSE, General Self Efficacy Scale; *denotes significant result at α = 0.05.

# denotes significant result upon accounting for the number of tests performed (α/10 = 0.005).

Repeated measures ANOVA revealed that higher scores were obtained at post-Recovery compared to post-COVID on AVLT1-5, AVLT6, and AVLT7 variables. Results are presented in Table 3.

**Table 3:** Post-COVID to post-recovery parametric repeated measures ANOVA results.

Variable	Time	*M* (*SD*)	Range	*Df*	*F*	η^2^	Sig. (*p*)	Cohen’s *d*
AVLT1-5	Post-COVID	7.29 (2.04)	[3.4, 12.4]	1	33.4	0.481	<0.0001*^#^	0.56
	Post-recovery	8.44 (2.04)	[4.2, 13.4]					
AVLT6	Post-COVID	6.46 (3.53)	[0, 14]	1	6.70	0.157	0.014*^#^	0.34
	Post-recovery	7.59 (3.35)	[0, 15]					
AVLT7	Post-COVID	6.11 (3.31)	[0, 14]	1	17.1	0.323	<0.001*^#^	0.46
	Post-recovery	7.78 (3.67)	[2, 15]					

AVLT, Auditory Verbal Learning Test (1–5 – average score of five learning repetitions, 6, score on the sixth repetition; 7, delayed recall score); Cohen’s *d* – effect size calculated as |postC-postR|/SDpostR; *denotes significant result at α = 0.05.

# denotes significant result upon accounting for the number of tests performed (α/3 = 0.017).

Table 4 provides a descriptive overview of the number of cases (%) that improved, remained unchanged, and worsened their score from post-COVID to post-Recovery for each variable. Additionally, at post-Recovery patients reported being at 90.1 (±14.9)% of their mental capacities (memory, concentration, etc.), and 88.9 (±20.4)% of their mood.

**Table 4:** Numbers and fractions of 2-month recovery rates.

Variable	# Cases improved (%)	# Cases unchanged (%)	# Cases worsened (%)
MoCA	22 (59.5)	8 (21.6)	7 (18.9)
sRT^1^	18 (48.6)	4 (10.8)	15 (40.5)
cRT^1^	17 (45.9)	3 (8.1)	17 (45.9)
CORSI	15 (40.5)	11 (29.7)	11 (29.8)
TMT-A1	22 (59.5)	2 (5.4)	13 (35.1)
TMT-B^2^	26 (70.3)	2 (5.4)	9 (24.3)
AVLT1-5	32 (86.5)	2 (5.5)	3 (8.1)
AVLT6	24 (64.9)	5 (13.5)	8 (21.6)
AVLT7	26 (70.3)	5 (13.5)	6 (16.2)
BDI	21 (56.8)	4 (10.8)	12 (32.4)
BAI	28 (75.7)	1 (2.7)	8 (21.6)
BC-CCI	14 (37.8)	9 (24.3)	14 (37.8)
GSE	14 (37.8)	5 (13.5)	18 (48.6)

See Tables 1, 2 for abbreviation.

1 If the score difference between the two time points in sRT and cRT was ≤5 ms, the results counted as unchanged.

2 If the score difference between the two time points in both TMT-A and TMT-B was ≤1 s, the result counted as unchanged.

### 3.2. Regression analysis

To investigate if the change in MoCA score from post-COVID to post-Recovery could be predicted, we calculated delta MoCA (ΔMoCA) as a relative difference between post-Recovery and post-COVID. Descriptive statistics of the predictors are displayed in Table 5.

**Table 5:** Descriptive statistics of regression predictors.

Regression predictors	*M* (*SD*)	Range
Comorbidity	n.a.	[0, 1]
FM [%]	30.2 (10.8)	[13.7, 58.2]
MEDLIFE DI	6.9 (2.25)	[3, 13]
Florida cognitive activities scale	30.8 (10.6)	[13, 58]

FM, Fat Mass; MEDLIFE DI, Mediterranean Lifestyle Diet Index; n.a., non-applicable.

A multiple linear regression predicting ΔMoCA in a stepwise forward fashion based on comorbidity, FM, MEDLIFE DI, and Florida Cognitive Activities Scale was significant but only for the comorbidity, while the FM, MEDLIFE DI, and Florida Cognitive Activities score were consequently excluded from the model. Regression analysis results are reported in Table 6.

**Table 6:** Results of regression analysis with surviving and excluded predictors.

Model: ΔMoCA	*R*	*R^2^*	*B*	Std error	Beta	*p*-value
Intercept			0.211	0.571		0.714
Included variable:						
Comorbidity	0.357	0.127	1.852	0.844	0.357	0.035*
Excluded variables:						
FM					0.109	0.518
MEDLIFE DI					−0.012	0.944
Florida cognitive activities scale					−0.015	0.927

Comorbidity, FM, MEDLIFE DI, and Florida Cognitive Activities Scale were entered into a stepwise regression model to predict the change in MoCA (ΔMoCA) score from post-COVID to post-Recovery; R, multiple correlation coefficient; *R*^2^, adjusted coefficient of determination, B, regression coefficient; beta – standardized regression coefficient. See Tables 2, 5 for abbreviation.

**Figure 1:**
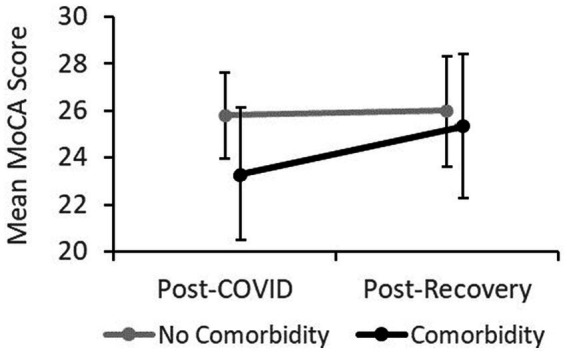
Mean MoCA score over time separated by comorbidity.

Participants’ predicted ΔMoCA was equal to 0.211 +  1.852*(comorbidity), where comorbidity was coded as 0 = “no comorbidity,” and 1 = “comorbidity.” Participants’ ΔMoCA increased 1.852 points when transitioning from no comorbidity to having at least one comorbidity. In “no comorbidity” group, ΔMoCA amounted to *M* = 0.211, *SD* = 1.686, while in the “comorbidity” group *M* = 2.056, *SD* = 3.019, and an independent samples *t*-test revealed that the two groups differed significantly (*t*(35) = −2.312, *p* = 0.027), suggesting greater change (increase) from post-COVID to post-Recovery in the “comorbidity” as opposed to the “no comorbidity” group. Figure 1 presents the mean MoCA scores separated by time and comorbidity.

## 4. Discussion

This study aimed to investigate cognitive functions and mood status in patients after hospitalization due to acute COVID-19 infection and at the 2-month follow-up. To our knowledge, this is the first attempt to assess the cognitive status following the recovery of the acute COVID-19 infection in the Slovenian population. Secondly, the study aimed to predict post-hospital cognitive recovery using four factors: comorbidity and FM, which have an established relationship with post-COVID recovery in other cultural backgrounds, and Mediterranean diet adherence and personal investment in everyday cognitive activities, whose relationship with the post-COVID recovery remains under-investigated.

Overall improvements from post-COVID to post-Recovery have been observed in global cognitive functioning as higher MoCA scores, as well as in specific cognitive domains, namely in the speed of visual search and executive function reflected by the faster TMT-A and TMT-B completion times, respectively, and the short-term memory and delayed recall observed in higher scores on auditory learning test measures. Additionally, from post-COVID to post-Recovery, depression and anxiety symptoms ameliorated in severity. Our results are in line with several studies that demonstrate acute COVID-19-related impairments on global cognitive function ranging from 15% (Van Den Borst et al., 2021) and up to 80% (Alemanno et al., 2021) of participants (for a review see Daroische et al., 2021), while the deficits reported in specific cognitive domains, such as executive function and short-term memory, have also been reported, however in smaller proportions (Almeria et al., 2020; Beaud et al., 2021; Negrini et al., 2021). Our study also confirmed evidence indicating that COVID-related cognitive impairment is typically accompanied by elevated levels of depression and anxiety and that both the cognitive symptoms and mood disturbances are typically at least partially recovered after several weeks or months following the post-acute COVID-19 period (Méndez et al., 2021; Pistarini et al., 2021). To gain insight into their subjective feeling of recovery progression, we asked the participants how many percent of their normal, pre-COVID state they currently stand at. On average, the subjective perception of the recovery was rated at approximately 90% for both mental capacity and mood, indicating that at 2-months post-COVID, the recovery felt to them as incomplete.

Despite significant recovery-related improvements, a considerable portion of participants demonstrated unchanged or even worsened cognitive performance at the 2-month follow-up; for the MoCA test, this portion amounted to 40.5% of participants. Also, 40.5% of participants demonstrated a MoCA score indicative of mild cognitive impairment. The aging effect caused by the elapsed 2-months is unlikely to explain the observed effect. A decline in the MoCA score of 1.7 points was reported over a period of 3.5 years in mildly cognitively impaired (MCI) individuals as opposed to the healthy ones (Krishnan et al., 2017), whereas in the present study in the 7 participants who experienced a decline, this amounted to 2.3 points in average. Similar to MoCA, the results of the AVLT test (35.1% for the AVLT6 and 29.7% for AVLT7) and TMT test (40.5% for TMT-A and 29.7% for the TMT-B) show comparable portions of participants in which improvement after recovery period did not occur. In the absence of the pre-COVID baseline scores we turned to the normative TMT (Tombaugh, 2004) and AVLT (Ivnik et al., 1990) scores which showed that after a 2-month recovery period, the percentages of participants scoring below the norms are consistently lower compared to that of the post-hospital discharge point, however approximately 45% and at least 75% of patients, respectively, still scored below the normative values after recovery and post-discharge. Although the norms were composed of participants with chronic illnesses, such as diabetes and hypertension, which makes their samples and our more alike and yields such medical comorbidities less likely to be the cause of the observed effects, it should be acknowledged that the fraction of patients with comorbidities in our sample is disproportionally high. We speculate that a more plausible explanation for the observed results indicates the presence of the post-acute sequelae or “Long COVID” – a chronic condition that develops during or after infection with SARS-CoV-2, ideally continues for more than 12 weeks and is not explained by an alternative diagnosis (NICE, 2021). The Long-COVID prevalence is estimated to develop in up to 25% of sufferers (Cirulli et al., 2020; Nehme et al., 2021; Sudre et al., 2021), and cognitive symptoms are among the most prevalent affecting approximately 70% of the Long-COVID patients (Cirulli et al., 2020; Bliddal et al., 2021). The design of the present study, however, does not allow making strong claims about the Long-COVID due to the insufficient duration of following the patients’ recovery.

The recovery did not seem to have any effect on the processing speed as demonstrated by the simple and choice RT task nor was the effect observed in the visuospatial working memory (CORSI) task. The subjective scales of cognitive complaints (BC-CCI) and general self-efficacy (GSE) also showed no difference between the two measurements, suggesting that the COVID-19 recovery was not associated with cognitive complaints or self-efficacy.

In the second part of the study, we aimed to predict the change in the MoCA from post-COVID to post-Recovery. Regression encompassed two predictors that show a relatively strong previously established relationship with disease severity/recovery, namely the comorbidity and fat mass, as well as two predictors whose relationship with recovery from COVID-19 is not that firmly established or has never been investigated, specifically the Mediterranean diet index and the engagement in cognitive daily activities, respectively. Against our expectations, comorbidity was the only variable that could significantly predict the change in MoCA score, while the fat mass, Mediterranean diet index, and Florida Cognitive Activities returned nonsignificant results. Literature suggests that people with medical comorbidities tend to suffer from more severe COVID-19 symptoms and higher fatality rates (Fu et al., 2020) and experience longer recovery times (Tolossa et al., 2021). Using machine learning algorithms, the symptom and comorbidity patient data alone proved able to predict the severity of the disease progression with a 90% accuracy (Chen et al., 2020). Our results are in line with the literature and indicate greater cognitive impairment at hospital discharge in the comorbidity group as opposed to the no-comorbidity group and show that almost a 2-point increase in MoCA score was expected in the comorbidity group compared to the no-comorbidity group following the 2-month recovery period. At the 2-month follow-up, the groups with and without comorbidity showed comparable mean scores, likely indicating that the no-comorbidity group suffered nonsignificant cognitive impairment at hospital discharge or that the recovery has not yet taken place as their overall mean scores remained relatively unchanged over the recovery period of 2 months. Our data indicate that the presence of comorbidity increases the risk of cognitive impairment upon COVID-19 infection. The comorbidity was validated as an important predictor of cognitive recovery following COVID-19 infection in a Slovenian sample. On the contrary, obesity and/or body-mass index have been implicated rather strongly in predicting COVID-19 severity/recovery (Cai et al., 2021; Jayanama et al., 2021) even in younger adults (Deng et al., 2020), however, results demonstrating no influence of body-mass index to recovery have also been observed (Abrahim et al., 2020). Mediterranean diet has been identified as a potential nutritional approach for COVID-19 due to its anti-inflammatory properties (Angelidi et al., 2021), however, in the present study, it showed no relationship with cognitive recovery. In the working population, Hershey et al. (2021) showed that individuals with a higher MEDLIFE index are less likely to experience metabolic syndrome. As we know that poorer metabolic health is an important factor associated with COVID-19 complications (Morys and Dagher, 2021), it would be worthwhile to further investigate this link between appropriate nutrition, metabolic health, and severe COVID-19 infections. Similarly, the cognitive daily activities investment, as assessed by the Florida Cognitive Activities Scale, during the recovery period did not predict cognitive recovery. Although the two predictors could be involved in the progression of and recovery from COVID-19, additional studies are needed to demonstrate their impact.

## 5. Limitations

It is important to acknowledge that the baseline data of the time before the COVID-19 infection was not obtained in this study, which poses a limitation to the conclusions regarding whether the recovery (if observed), was complete at the 2-month follow-up investigation. In other words, we are blind to whether the level of cognitive functioning returned to the level before the infection. The within-subject design of the study allowed exclusively to make claims about the cognitive function and mood changes related to the recovery period. Secondly, all cognitive tests were repeated at a 2-month follow-up, which allows for the learning effect to occur. The original MoCA validation study reported a 0.91 test–retest consistency at 2 months with no significant learning effect (Julayanont et al., 2015), however, the indication of improved performance following repeated MoCA application (Cooley et al., 2015) stresses the importance of interpreting the results with caution. The learning and recovery effects could have been addressed more adequately upon the inclusion of the control group. Thirdly, the influence of sedating and anesthetic drugs given to the patients while hospitalized (without detailed dosage and duration records) might have contributed to cognitive impairments at the first measurement time point and biased the results in favor of the post-Recovery time point. Nevertheless, if this was true, the comorbidity and no-comorbidity groups should have been affected in a similar fashion, which is not the case. Also, the first measurement time point was performed on the 10^th^ day after the last negative swab allowing sufficient time for drug clearance. Fourthly, the normative data considered for the TMT and AVLT tests were not computed on a Slovenian sample but belonged to the populations of Canada and Minnesota (US), respectively. Also, the normative data taken into consideration belonged to a group of 55–59 years old, which captured the mean age of the patients in the present study (*M* = 58,2), however, our subjects belonged to a rather heterogeneous age group spanning over a wide range of 34 to 79 years old. Comparison to normative data could be misleading also because the studied sample was not composed via random selection and might therefore suffer from the lack of generalizability to the broader population. Despite the convenience sampling approach, our sample remained relatively small and of limited diversity, suggesting that interpretation of the results be performed with care.

## 6. Conclusions

The results of this study show impairment of the global cognitive function, executive function, memory, and mood in patients after acute COVID-19 recovery. At 2-month post-hospital discharge, the assessment of the global cognitive function shows significant improvement and scores above the cognitive impairment threshold have been observed in almost 60% of patients. Out of the remaining 40% of participants, some could be suffering from the Long-COVID syndrome, however, the exact estimates are difficult to make. Comorbidity was validated as an important predictor of cognitive recovery following COVID-19 infection. To adequately assess the rates of cognitive recovery over time, new studies should adopt a longitudinal study design while controlling for the practice effect by employing alternative versions of the used tests; for example, such versions have been developed for the MoCA (Lebedeva et al., 2016) and TMT (Wagner et al., 2011).

## Data availability statement

The raw data supporting the conclusions of this article will be made available by the authors, without undue reservation.

## Ethics statement

The studies involving human participants were reviewed and approved by Ethical committee of the General Hospital Izola (application number: 1/21). The patients/participants provided their written informed consent to participate in this study.

## Author contributions

RP and MG designed the study protocol while MP and UM implemented the cognitive and psychological measures. MP, BŠ, LŠ, SP, KT, and UM collected the data. MP analyzed the data and drafted the manuscript while BŠ advised on data analysis. All authors provided commentary and critical revisions to the draft, and approved the final version of the manuscript for publication.

## Funding

This study was supported by the Slovenian Research Agency (research core Funding No. P5-0381). MP and UM also acknowledge financial support from the European Union’s Horizon 2020 research and innovation program under grant agreement No. 952401 (TwinBrain – TWINning the BRAIN with machine learning for neuro-muscular efficiency).

## Conflict of interest

The authors declare that the research was conducted in the absence of any commercial or financial relationships that could be construed as a potential conflict of interest.

## Publisher’s note

All claims expressed in this article are solely those of the authors and do not necessarily represent those of their affiliated organizations, or those of the publisher, the editors and the reviewers. Any product that may be evaluated in this article, or claim that may be made by its manufacturer, is not guaranteed or endorsed by the publisher.
