# Supplementary material for: Effects of COVID-19 on cognition and mood after hospitalization and at 2-month follow-up
Source: Front Psychol. 2023 May 26;14:1141809. doi: 10.3389/fpsyg.2023.1141809 (PMC10252562; doi:10.3389/fpsyg.2023.1141809)
Supplement: Supplementary file 1 [file Table_1.docx]

SUPPLEMENTARY MATERIAL

Table S1 Fraction of patients with scores below normative values or expressing cognitive impairment or at least mild mood symptoms severity at both post-COVID and post-Recovery.

|  |  | Fraction of patients (%) | |
| --- | --- | --- | --- |
| Measure | Impairment Criteria | Post-COVID | Post-Recovery |
| MoCA | < 26 points | 59.5 | 40.5 |
| TMT-A | > 35.1 seconds | 48.6 | 43.2 |
| TMT-B | > 78.8 seconds | 54.1 | 45.9 |
| AVLT1-5 | < 10.4 points | 89.2 | 75.7 |
| AVLT6 | < 11.2 points | 91.9 | 89.2 |
| AVLT7 | < 10.4 points | 89.2 | 73 |
| BDI | 11 – 30 points: Mild-to-moderate depression | 29.7 | 24.3 |
| BAI | 8 – 25 points: Mild-to-moderate anxiety  26 – 63 points: Severe anxiety | 37.8  8.1 | 24.3  5.4 |

For abbreviation see Table 2.
